# Patterns of patients with polypharmacy in adult population from Korea

**DOI:** 10.1038/s41598-022-23032-z

**Published:** 2022-10-27

**Authors:** Woo-young Shin, Tae-Hwa Go, Dae Ryong Kang, Sei Young Lee, Won Lee, Seonah Kim, Jiewon Lee, Jung-ha Kim

**Affiliations:** 1 Department of Family Medicine, Chung-Ang University College of Medicine, Seoul, Republic of Korea; 2 Department of Biostatistics, Yonsei University Wonju College of Medicine, Wonju, Republic of Korea; 3 Department of Precision Medicine, Yonsei University Wonju College of Medicine, Wonju, Republic of Korea; 4 Department of Otorhinolaryngology-Head and Neck Surgery, Chung-Ang University College of Medicine, Seoul, Republic of Korea; 5 Department of Nursing, Chung-Ang University, Seoul, Republic of Korea; 6 Department of Family Medicine, Health Promotion Center, Chung-Ang University Hospital, Seoul, Republic of Korea; 7 Department of Family Medicine, Chung-Ang University College of Medicine, 102 Heukseok-ro, Dongjak-gu, Seoul, 06973 Republic of Korea

**Keywords:** Health care, Medical research

## Abstract

Polypharmacy and its rising global prevalence is a growing public health burden. Using a large representative nationwide Korean cohort (N = 761,145), we conducted a retrospective cross-sectional study aiming to identify subpopulations of patients with polypharmacy and characterize their unique patterns through cluster analysis. Patients aged ≥ 30 years who were prescribed at least one medication between 2014 and 2018 were included in our study. Six clusters were identified: cluster 1 mostly included patients who were hospitalized for a long time (4.3 ± 5.3 days); cluster 2 consisted of patients with disabilities (100.0%) and had the highest mean number of prescription drugs (7.7 ± 2.8 medications); cluster 3 was a group of low-income patients (99.9%); cluster 4 was a group of high-income patients (80.2%) who frequently (46.4 ± 25.9 days) visited hospitals/clinics (7.3 ± 2.7 places); cluster 5 was mostly elderly (74.9 ± 9.8 years) females (80.3%); and cluster 6 comprised mostly middle-aged (56.4 ± 1.5 years) males (88.6%) (all *P* < 0.001). Patients in clusters 1–5 had more prescribed medications and outpatient visit days than those in cluster 6 (all *P* < 0.001). Given limited health care resources, individuals with any of the identified phenotypes may be preferential candidates for participation in intervention programs for optimal medication use.

## Introduction

Polypharmacy is a growing major public health burden with rising global prevalence; the proportion of the older population continues to increase and they usually have a number of chronic health conditions^1^. Although there is no clear consensus on the definition of polypharmacy, the term is commonly defined as the routine use of five or more concurrent medications^2–4^. It has been estimated that the global prevalence of polypharmacy among residents in long-term care facilities accounts for 38–91%^5^. In Korea, approximately half of the elderly population, aged ≥ 65 years, receive multi-drug prescriptions and considering the current aging trend, that number is expected to further increase in the future^6,7^.

Patients with polypharmacy have a higher risk of harmful effects, such as medication errors, adverse drug reactions, falls, dizziness, and increases in hospitalization and mortality^8–10^. It has been reported that inappropriate management of polypharmacy has a significant impact on avoidable expenditure on health care resources and costs, leading to a large economic burden^11,12^. Accordingly, the World Health Organization has asked countries and the concerned authorities to prioritize medication safety in polypharmacy, take early action, and reduce avoidable medication-related harm^13^.

Although some countries are developing evidence-based strategies and introducing them for the optimal use of multiple medications, structured management programs or their supporting policies are still limited in many countries, including Korea^14–18^. It is emphasized that a comprehensive consideration in the clinical context, rather than a simple approach with the number of medications used, is essential to develop rational policies to improve polypharmacy use behavior^2,3,19^. To the best of our knowledge, most studies on polypharmacy conducted in Korea have only targeted certain vulnerable patient groups, such as the elderly and severely ill, and only simple status, including the prevalence of polypharmacy and its mortality, have been reported^6,7,20^. In-depth research to determine several distinct phenotypes of patients with polypharmacy could be helpful in developing effective management strategies by diversifying their countermeasures accordingly.

Therefore, using a large nationwide cohort of Korean adults, this study aimed to identify subpopulations of patients with polypharmacy and characterize their unique patterns through cluster analysis.

## Results

A total of 761,145 patients were included in the training set for cluster analysis. Their mean age was 67.05 ± 12.57 years, and those aged 70–79 years accounted for majority (29.78%). Of all patients, 88,674 (11.65%) were medical aid beneficiaries. The mean number of outpatient visits was 30.61 ± 27.11 days, and 226,283 patients (29.73%) were hospitalized. Among medical institutions by type, the medical clinic was visited by most patients (89.84%). Patients visited a mean of 4.72 ± 2.87 medical institutions per year and prescribed 7.03 ± 2.42 medications. The most common diseases among patients were chronic gastritis/gastroesophageal reflux disease (639,017 patients, 83.95%), hypertension (584,156 patients, 76.75%), and dyslipidemia (503,941 patients, 66.21%) (Table 1).

**Table 1:** Characteristics of study patients with polypharmacy in 2014–2018 (n = 761,145).

Variable	Patients with polypharmacy^a^	
	Number	%
**Sex**		
Male	368,994	48.48
Female	392,151	51.52
**Age**	67.05 ± 12.57	
30–39 years	17,495	2.30
40–49 years	52,981	6.96
50–59 years	139,280	18.30
60–69 years	199,274	26.18
70–79 years	226,686	29.78
80–89 years	112,250	14.75
≥ 90 years	13,179	1.73
**Type of medical coverage**		
National Health Insurance subscriber	672,471	88.35
Medical aid beneficiary	88,674	11.65
**Income level**		
Medical aid beneficiary	88,674	11.65
Quarter 1	128,578	16.89
Quarter 2	117,658	15.46
Quarter 3	162,679	21.37
Quarter 4	263,556	34.63
**Disabled**	161,230	21.18
**Number of visit days per year: outpatient**	30.61 ± 27.11	
0–10 days	105,774	13.90
11–20 days	223,451	29.36
21–30 days	163,195	21.44
≥ 31 days	268,725	35.31
**Hospitalization experience**	226,283	29.73
**Number of hospitalization days per year: inpatient**	0.84 ± 2.42	
No hospitalization	534,862	70.27
1–5 days	200,000	26.28
≥ 6 days	26,283	3.45
**Visited medical institute**		
Medical clinic	683,823	89.84
Hospital	261,544	34.36
General hospital	362,682	47.65
Tertiary hospital	251,471	33.04
Long-term care hospital	29,689	3.90
**Number of visited medical institute per year**	4.72 ± 2.87	
1 place	66,383	8.72
2–5 places	447,531	58.80
≥ 6 places	247,231	32.48
**Number of prescribed medications per year**	7.03 ± 2.42	
**Diagnosed disease**		
Hypertension	584,156	76.75
Dyslipidemia	503,941	66.21
Knee arthrosis	242,638	31.88
Diabetes mellitus	381,482	50.12
Chronic ischemic heart disease	201,807	26.51
Liver disease	186,081	24.45
Depression	140,630	18.48
Asthma/chronic obstructive pulmonary disease	250,468	32.91
Osteoporosis	144,984	19.05
Kidney disease	35,717	4.69
Chronic stroke	149,938	19.70
Dementia	93,943	12.34
Anxiety disorder	170,648	22.42
Parkinson’s disease	29,073	3.82
Cancer	71,217	9.36
Chronic gastritis/gastroesophageal reflux disease	639,017	83.95

^a^Categorical variables are expressed as frequencies and percentages, and continuous variables are expressed as mean values with standard deviation.

**Figure 1:**
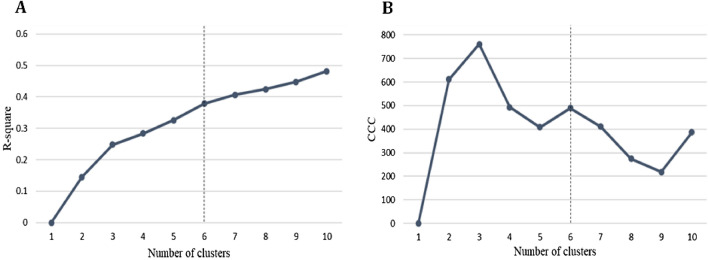
Scree plots for the K-means cluster analysis of study patients with polypharmacy. The elbow method was performed to determine the optimal number of clusters by estimating the (**A**) R-squared value and (**B**) CCC according to the number of clusters. The analysis used variables including sex, age, type of medical coverage, income level, disability, number of outpatient visits or hospitalization days per year, and number of visited medical institutes per year. These plots indicate that K = 6 is the optimal number of clusters in this study. *CCC* cubic clustering criterion.

**Figure 2:**
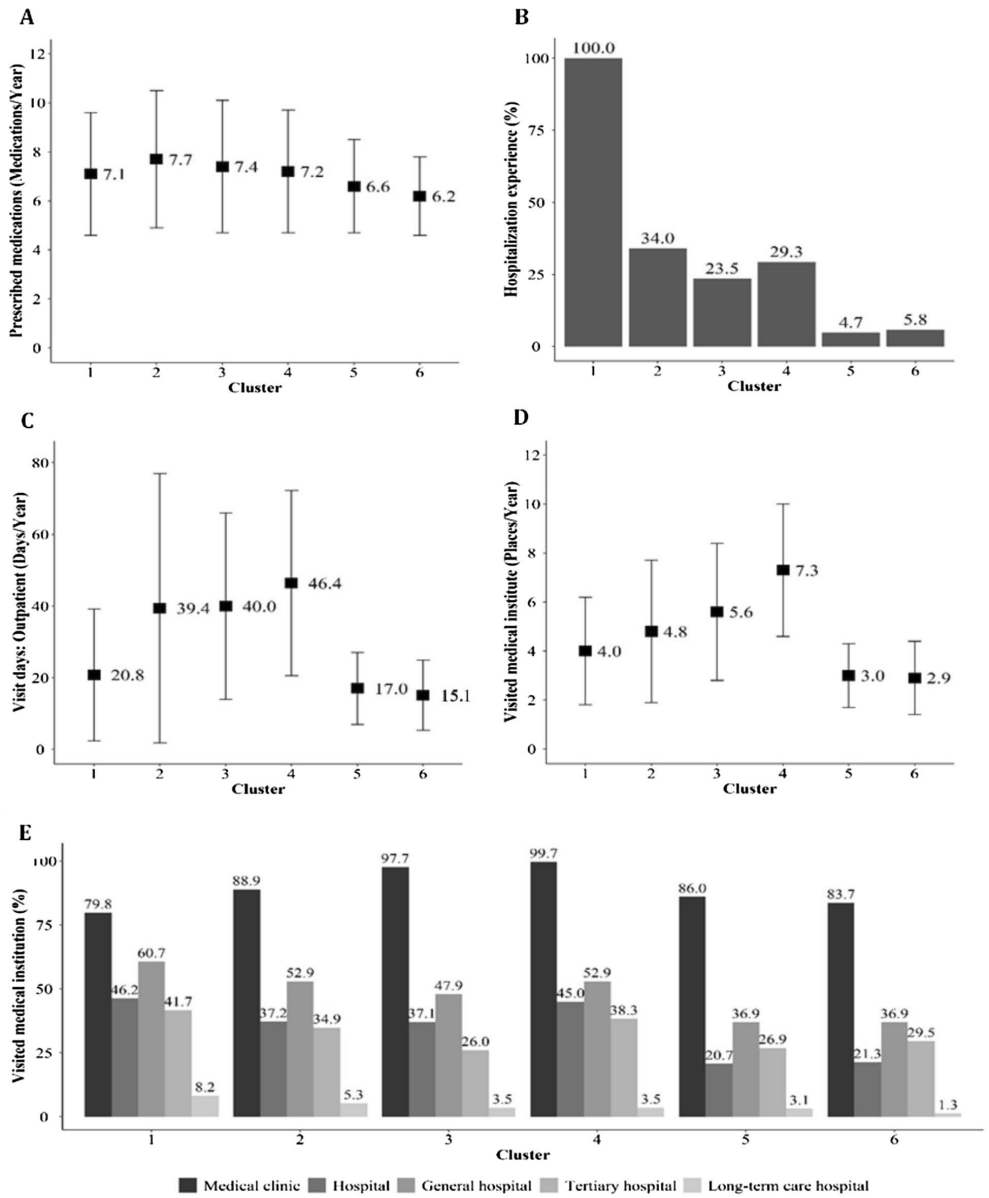
Comparison of health care utilization in six clusters of study patients with polypharmacy. Categorical variables were evaluated by chi-square test and continuous variables by t-test to compare the characteristics of each cluster. The following attributes are described by cluster: (**A**) the number of prescribed medications per year; (**B**) hospitalization experience; (**C**) the number of outpatients visit days per year; (**D**) the number of medical institutes visited by patients per year; and (**E**) medical institution type visited by patients (all *P* < 0.001).

**Figure 3:**
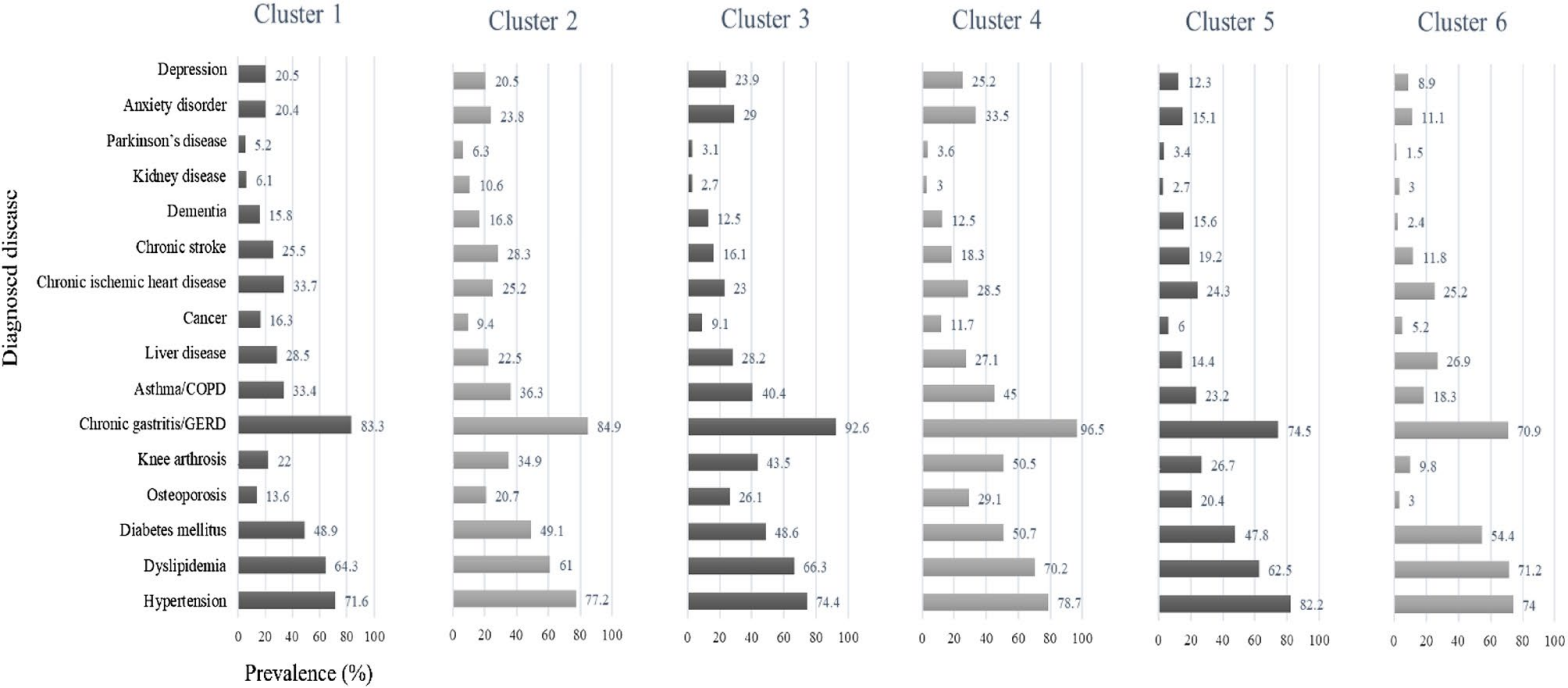
Comparison of diagnosed diseases in six clusters of study patients with polypharmacy. To compare the diagnosed diseases of patients in each cluster, they were evaluated by chi-square test and described by cluster (all *P* < 0.001). *GERD* gastroesophageal reflux disease, *COPD* chronic obstructive pulmonary disease.

The results of determining the optimal number of clusters, using the elbow method, are presented in Fig. 1. The scree plots provided the optimal number of clusters, which was determined to be six. Of the six clusters of patients with polypharmacy, cluster 4 had the largest distribution. In cluster 5, the proportion of females was 80.3%, and the average age was the highest, at 74.9 years. In cluster 6, 88.6% were male, and the average age was 56.4 years. In cluster 3, 44.5% were medical aid beneficiaries, and the remaining 55.4% were National Health Insurance subscribers with relatively low incomes. On the other hand, there was no medical aid beneficiary in cluster 4 and 80.2% had a relatively high income level. All patients in cluster 2 had disabilities (all *P* < 0.001) (Table 2). The characteristics of health care utilization by cluster are shown in Fig. 2. Cluster 2 had the highest number of prescribed medications (7.7 ± 2.8 medications). In cluster 1, all patients experienced hospitalization, and 27.7% of the patients were hospitalized for > 6 days, which was the highest among the clusters. The proportion of outpatient visits exceeding 31 days per year was 77.5% in cluster 4, which was significantly higher than that of the other clusters. As for the number of visiting medical institutions, 80.8% of cluster 4 visited six or more institutions per year (all *P* < 0.001) (Fig. 2). The results of the multiple linear regression analysis are presented in Supplementary Table S1. Compared with patients in cluster 6, those in clusters 1–5 reported more prescribed medications (β = 0.70, standard error = 0.01; 1.10, 0.01; 0.65, 0.01; 0.76, 0.01; and 0.09, 0.01, respectively) and more outpatient visit days (β = 5.28, standard error = 0.11; 23.67, 0.10; 24.23, 0.12; 30.69, 0.10; and 0.96, 0.11, respectively) (all *P* < 0.001) (Supplementary Table S1). Regarding diagnosed diseases, cluster 1 had a higher cancer prevalence than other clusters (16.3%) and a higher number of severe diseases. The prevalence of hypertension was 82.2% in cluster 5, that of dyslipidemia was 71.2% in cluster 6, and that of diabetes was 54.4%, which was higher than that of other clusters (all *P* < 0.001) (Fig. 3). The most frequently prescribed medications in every cluster were the same in the order of aspirin, atorvastatin, and metformin (data not shown).

**Table 2:** Sociodemographic patterns of patients with polypharmacy using K-means clustering (n = 761,145).

Variable	Cluster 1	Cluster 2	Cluster 3	Cluster 4	Cluster 5	Cluster 6	*P* value^a^
**Prevalence**	93,074 (12.2)	139,975 (18.4)	95,004 (12.5)	168,148 (22.0)	125,732 (16.5)	139,212 (18.3)	
**Sex**							< 0.001
Male	58,158 (62.5)	70,988 (50.7)	29,498 (31.1)	62,307 (37.1)	24,745 (19.7)	123,298 (88.6)	
Female	34,916 (37.5)	68,987 (49.3)	65,506 (69.0)	105,841 (63.0)	100,987 (80.3)	15,914 (11.4)	
**Age**	64.3 ± 12.8	68.6 ± 12.0	67.0 ± 12.0	70.3 ± 10.2	74.9 ± 9.8	56.4 ± 1.5	< 0.001
30–39 years	2788 (3.0)	2741 (2.0)	1637 (1.7)	1343 (0.8)	0 (0.0)	8986 (6.5)	
40–49 years	8935 (9.6)	7518 (5.4)	5571 (5.9)	4706 (2.8)	220 (0.2)	26,031 (18.7)	
50–59 years	22,230 (23.9)	20,597 (14.7)	19,005 (20.0)	18,283 (10.9)	8406 (6.7)	50,759 (36.5)	
60–69 years	25,573 (27.5)	35,033 (25.0)	27,254 (28.7)	44,396 (26.4)	25,574 (20.3)	41,444 (29.8)	
70–79 years	21,993 (23.6)	49,206 (35.2)	26,536 (27.9)	71,226 (42.4)	46,967 (37.4)	10,758 (7.7)	
80–89 years	10,521 (11.3)	22,807 (16.3)	13,662 (14.4)	26,513 (15.8)	37,513 (29.8)	1234 (0.9)	
≥ 90 years	1034 (1.1)	2073 (1.5)	1339 (1.4)	1681 (1.0)	7052 (5.6)	0 (0.0)	
**Type of medical coverage**							< 0.001
National health insurance subscriber	82,321 (88.4)	108,737 (77.6)	52,726 (55.5)	168,148 (100.0)	122,854 (97.7)	137,685 (98.9)	
Medical aid beneficiary	10,753 (11.6)	31,238 (22.4)	42,278 (44.5)	0 (0.0)	2878 (2.3)	1527 (1.1)	
**Income level**							< 0.001
Quarter 1	16,982 (18.3)	22,374 (16.0)	39,020 (41.1)	11,345 (6.8)	14,560 (11.6)	24,297 (17.5)	
Quarter 2	16,862 (18.1)	17,842 (12.8)	13,571 (14.3)	21,846 (13.0)	17,382 (13.8)	30,155 (21.7)	
Quarter 3	21,180 (22.8)	25,586 (18.3)	135 (0.1)	47,827 (28.4)	30,530 (24.3)	37,421 (26.9)	
Quarter 4	27,297 (29.3)	42,935 (30.7)	0 (0.0)	87,130 (51.8)	60,382 (48.0)	45,812 (32.9)	
**Disabled**	13,231 (14.2)	139,975 (100.0)	13 (0.0)	35 (0.0)	2282 (1.8)	5694 (4.1)	< 0.001

Categorical variables were evaluated using the chi-square test as frequencies (percentages), and continuous variables by t-test as mean values with standard deviation.

^a^*P* values < 0.05 were statistically significant.

Variables with O/E ratios of ≥ 2 or exclusivity of ≥ 25% were observed in each cluster (Supplementary Table S2). The highest O/E ratio and exclusivity were hospitalization for ≥ 6 days in cluster 1 (8.02% and 98.03%, respectively) (all *P* < 0.001). The validity of this clustering was confirmed by randomly selecting the patients as a test set.

## Discussion

To our knowledge, this is the first study to determine subpopulations of patients with polypharmacy and characterize their patterns in a large nationwide cohort. We identified six distinct phenotypes of patients with polypharmacy in the Korean adult population.

In patients with polypharmacy, cluster 1 mostly included patients with severe disease who were hospitalized for a long time or frequently. Cluster 2 was patients with disabilities who had many inpatient and outpatient visits and the highest average number of prescription drugs among all clusters. Cluster 3 was low-income patients, including medical aid beneficiaries, who frequently visited several clinics, while cluster 4 was high-income patients who visited multiple clinics most frequently. Cluster 5 was mostly elderly females, whereas cluster 6 was mostly middle-aged males. Most patients in these last two clusters had multiple mild chronic diseases, but the frequency of visits to medical institutions was relatively lower than in other clusters.

There have been several studies on the characteristics of patients with polypharmacy or related factors. However, no clustering studies have been conducted on polypharmacy based on multi-faceted patient factors. Clustering has usually been performed in a single disease to identify its subtypes of patients regarding their prognosis and to provide tailored approaches to those subtypes^21–23^. Cluster analysis on multimorbidity patients was performed, but only clustering of frequently morbid diseases or prescription drugs was performed^24^. Our study identified some characteristic phenotypes in patients with polypharmacy, including sociodemographic factors, clinical status, and medical utilization behavior, through K-means cluster analysis.

It has been well elucidated that older age, living in care homes, cancer survivors, and multimorbidity are significantly associated with higher prevalence of polypharmacy^3,25^. In their study in Japan, Ishizaki et al. reported that patients with more hospitalizations and visits to medical institutions were at a higher risk of polypharmacy^26^. In our study, most patients with polypharmacy were elderly people with multimorbidity and a group comprising inpatients with severe diseases, such as cancer, was also included. However, in one cluster, the majority were middle-aged males and the patients in some clusters were those with mild chronic diseases who had relatively few visits to medical institutions and did not receive sufficient health care. A study conducted in the United States reported a significant relationship between polypharmacy in Medicaid beneficiaries and higher medical use and expenditures^27^. This is in line with one cluster found in our study. In Korea, the medical aid program, a public assistance system similar to Medicaid in the United States, has been operated by government authorities for low-income people. Health care services are provided to the entire population in Korea based on a national fee-for-service and mainly two types of health security systems are in operation^28^. The National Health Insurance program, which is a compulsory social insurance, allows the insured’s contributions and the government subsidy to share health care costs^28^. Some low-income households are guaranteed to receive appropriate medical services without financial difficulties as medical aid beneficiaries^29^. The patients in cluster 3 consisted of low-income people with relatively frequent visits to several clinics in the community; many medical aid beneficiaries were included. However, the patients in cluster 4 were high-income older people who used multiple clinics most frequently (Table 2, Fig. 2). In both clusters, the patients often had psychiatric and musculoskeletal disorders and asthma or chronic obstructive pulmonary disease (Fig. 3).

Interestingly, we found that the types of patients with polypharmacy included these opposing groups: inpatients with severe disease or disability, outpatients with mild chronic disease, medical aid beneficiaries and patients with high-income level, and patients who overuse health care and those who do not even receive essential medical care. These findings support that a multi-faceted approach to manage polypharmacy would be more effective in ensuring the optimization of medication prescriptions in patients with various characteristics, rather than a single strategy. Although the policies and systems for polypharmacy management in Korea are still in the introductory stage and mainly focused on general drug safety management, several programs that have demonstrated significant clinical effects in optimizing medication use in patients with polypharmacy have been implemented in other countries^30–32^. These programs commonly provide systematic and structured medication reviews to patients, usually the elderly with multimorbidity. They presented guidelines for patient-centered clinical polypharmacy management with a comprehensive evaluation and approach, rather than simply reducing the number of medications prescribed^30–32^. According to the results of this study, it would be more efficient to diversify intervention strategies by target groups for patient-centered management. For example, hospital-based programs that provide polypharmacy management in hospital-level medical institutions may be appropriate for patients with severe chronic diseases or disabilities. The multidisciplinary team approach of medication management would be effective in the case of a hospital-based model. A clinic-based model in the community would be appropriate for low-income or high-income elderly patients with relatively more frequent visits to multiple clinics. It may be effective to designate regularly visited clinics and activate the primary health care system to control the number of indiscriminate visits to medical institutions. It has been noted that psychotropic drugs including benzodiazepines and antidepressants are one of the most commonly over-prescribed medications worldwide^33,34^. Previous study has also shown that excessive use of Nonsteroidal anti-inflammatory drugs or overtreatment in patients with musculoskeletal diseases such as arthritis and osteoporosis are common in clinical practice^35,36^. Based on our findings, it is necessary to manage over-prescription of psychiatric and nonsteroidal anti-inflammatory drugs since patients with polypharmacy often have psychiatric and musculoskeletal disorders. It is also necessary to reorganize the health system to prevent doctor shopping and duplicate prescriptions targeting them as patients often have psychiatric diseases, low-income medical aid beneficiaries included. Elderly females and middle-aged males with mild multimorbidity did not have a relatively high number of visits to medical institutions or prescription medications. Clinic-based programs may be appropriate for patients with mild multimorbidity. It would be effective to provide adequate chronic disease management in primary health care settings. It is necessary to develop and apply standardized management guidelines for multimorbidity, rather than only a single disease.

Given the limited health care resources, it would be efficient for individuals with any of the phenotypes identified in our study to preferentially be included as candidates for participation in intervention programs for optimal medication use.

This study has several limitations that must be carefully considered when interpreting the results. The data used in this study were collected by the National Health Insurance Service (NHIS) for the purposes of claim and reimbursement of medical service costs, not for research purposes^37^. These collected data did not include records of purchases of non-reimbursable prescriptions and over-the-counter medications that are not covered by the National Health Insurance. However, it is expected to reflect real-world clinical practice through the large sample size, which is nationally representative^37^. Based on our medication coding method, the number of all medication ingredients that were administered may be greater than the estimated number. The K-means clustering method is not robust to some outliers. Outliers are data that are very far from the cluster centroid and other data points^38^. Thus, further in-depth studies on the detailed factors related to polypharmacy are needed in the future. Finally, our results were obtained in the Korean health care delivery system. Therefore, they are not generalizable worldwide and consideration of local circumstances is required.

Despite these limitations, this study systematically identified novel patterns and types of patients with polypharmacy and provides a basis for developing effective management programs for tailored improvement of their medication use behavior. Furthermore, our findings could be helpful in developing effective multi-faceted strategies or their supporting policies for the optimal use of multiple medications in patients with various characteristics in the current Korean healthcare system.

In conclusion, this study elucidates distinct subpopulations in terms of sociodemographics, clinical features, and health care utilization in patients with polypharmacy. These findings could contribute to reducing the burden of inappropriate polypharmacy and facilitate appropriate medication use by developing tailored strategies for patients with different tendencies and characteristics.

## Methods

### Study design and data source

**Figure 4:**
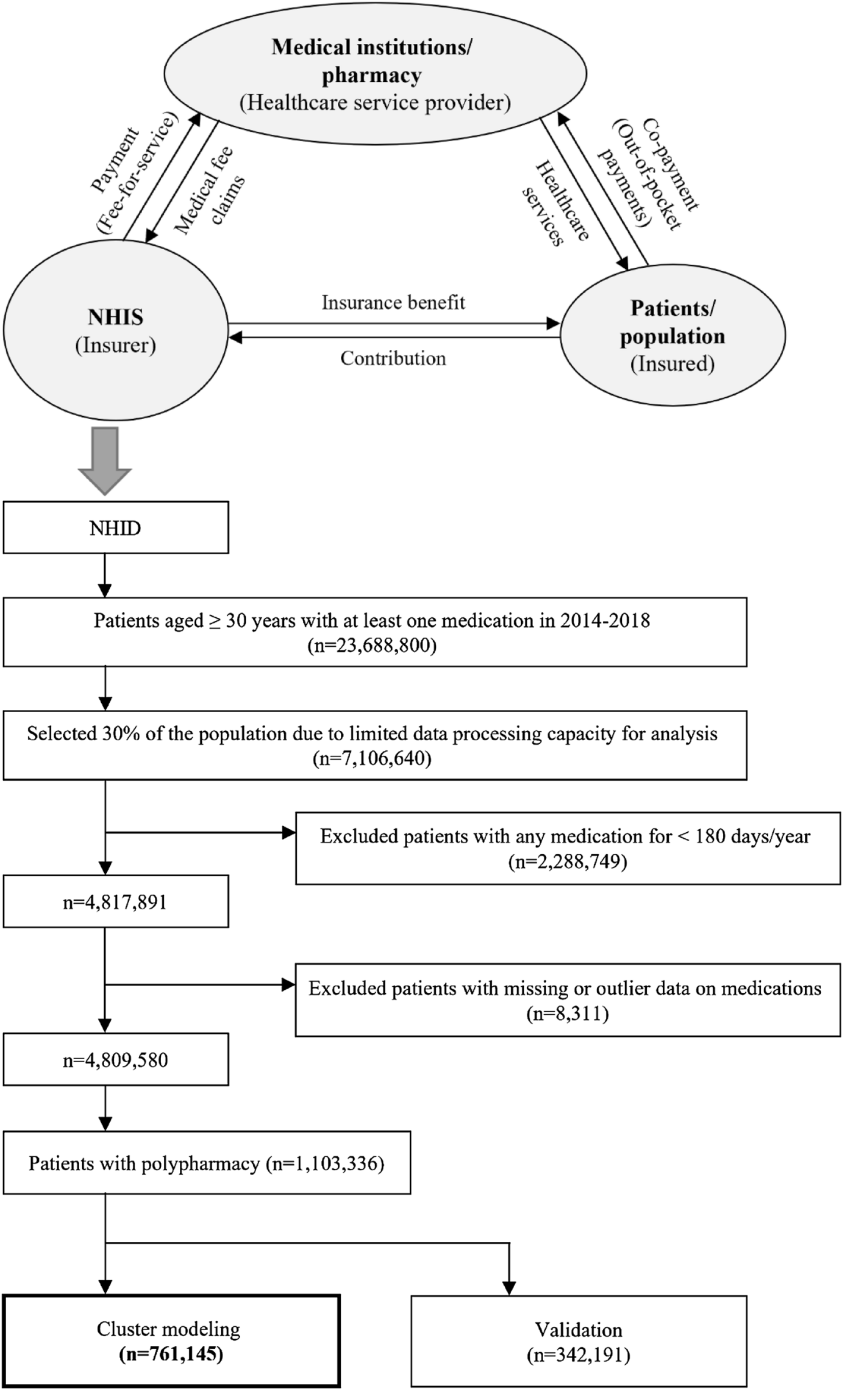
The structure of the National Health Insurance program in Korea and this study population data obtained from the NHID. *NHID* National Health Information database, *NHIS* National Health Insurance Service.

This was a retrospective cross-sectional study, performed using a non-hierarchical clustering method. We used data from the National Health Information Database (NHID), a nationwide database of the entire Korean population. The NHID is a public database with a large volume of health insurance information, including sociodemographics, insurance eligibility, treatment details, medical institution status, and health care utilization among the entire Korean population^37^. Almost all citizens in Korea are obliged to join the National Health Insurance program as part of the social security system^39^. The NHIS constructs the NHID by collecting medical information of all citizens based on the health care delivery system of the fee-for-service model and provides it as de-identified data to support various research activities^37^ (Fig. 4).

This study was conducted in accordance with the Declaration of Helsinki. Written informed consent was waived by the institutional review board of Chung-ang University Hospital because of the characteristics of NHIS data. Before we used the data from the NHID, this study was approved by the Institutional Review Board (IRB) of Chung-ang University Hospital (IRB no.: 2003-010-19308).

### Coding of medications

When coding medications in this study, we only used the data on oral medications prescribed to outpatients or inpatients. To clarify the number of prescribed medications, we excluded injections and topical drugs. Medications prescribed for < 180 days per year were also excluded to not include cases of temporary polypharmacy status during the analysis. Each medication was coded based on its main ingredients.

### Definition of polypharmacy

Polypharmacy was defined as a case in which five or more medications with different main ingredients were used simultaneously, among prescribed medications. However, over-the-counter medications or medications not covered by health insurance were not included in this definition.

### Study Population

Patients aged ≥ 30 years who were prescribed at least one medication from January 1, 2014, to December 31, 2018, were initially retrieved from the entire Korean population enrolled in the NHID. Due to the limited data processing capacity of the server for analysis, 30% of the population (7,106,640 patients) were obtained using a simple random sampling method. We excluded 2,288,749 patients who were prescribed any medication for < 180 days per year to not include cases of temporary polypharmacy status during the analysis. We also excluded patients whose data on prescribed medications were missing or outliers. Of the remaining 4,809,580 patients, those who had five or more medications prescribed simultaneously were included in this study (1,103,336 patients).

A total of 761,145 patients were finally included for cluster modeling as a training set (70% with random sampling), after excluding patients in the test set (remaining 30%) for validation (Fig. 4).

### Variables in clustering

Data on sociodemographic characteristics (sex, age, type of medical coverage, income level, and disability), health care utilization (number of outpatient/hospitalization days per year and medical institutions visited), and clinical information (prescribed medications and diagnosed diseases) were collected during the study period.

The study patients were classified into two types according to their medical coverage in Korea. Most of the Korean population is obligated to join the National Health Insurance, so their medical expenses are shared with the government^28^. Some other populations are included in the government medical aid program and are guaranteed to receive appropriate medical services without financial difficulties^29^. These medical aid beneficiaries include low-income populations who cannot afford to pay for health care expenses. In this study, except medical aid beneficiaries, the remaining National Health Insurance subscribers were divided into quartiles from the lowest (quartile 1) to the highest income (quartile 4). The number of patients prescribed medication, medical institutions visited, and number of outpatient/hospitalization days were counted on an annual basis. Medical institutions visited by patients were classified by type according to the number of beds and difficulty of treatment, based on the Korean Medical Law. A medical clinic is a medical institution that provides medical treatment to outpatients for common diseases that are frequently encountered in daily life and provides comprehensive health care services through initial contact with patients and can accommodate up to 29 beds^40^. A hospital was defined as a medical institution with > 30 and < 100 beds, mainly providing medical care to inpatients^40^. A general hospital mainly provides medical care to inpatients, with > 100 beds, > 7 departments, and an exclusive specialist for each department^40^. A tertiary hospital is a general hospital that specializes in high-difficulty treatment for severe diseases and is designated after deliberation by health authorities^40^. The tertiary hospital has > 20 medical departments and specialists dedicated to each department. A long-term care hospital is defined as a hospital with ≥ 30 beds for patients requiring long-term hospitalization. This hospital is mainly for patients who are geriatric, chronically ill, and recovering after surgical operations or injuries^40^. When a diagnosed disease is coded for each patient from the NHID, the prevalence is not estimated based on the number of related prescriptions received each year, and the information on the five main diseases registered to each patient from any medical institution is retrieved and classified according to the International Classification of Diseases version 10^37^. The inpatient/outpatient prescription medication history was also collected.

### Statistical analysis

For all analysis results, categorical variables are expressed as frequencies and percentages and continuous variables as mean ± standard deviation.

We conducted a descriptive analysis of the study population. K-means cluster analysis was performed to define the clusters of study patients with polypharmacy, and the SAS FASTCLUS procedure was applied using the following variables: sex, age, type of medical coverage, income level, disability, number of outpatient visits or hospitalization days per year, and number of visited medical institutes per year. These variables showed significant differences between polypharmacy (1,103,336 patients) and non-polypharmacy patients (3,706,244 patients) in the initially obtained study population (4,809,580 patients) (data not shown). The R-squared value and cubic clustering criterion (CCC), according to the number of clusters, were estimated to determine the optimal number of clusters, using the elbow method. To compare the characteristics of each cluster obtained, categorical variables were evaluated using the chi-square test and continuous variables by t-test. Multiple linear regression analysis was conducted to compare health care utilization in the six clusters after adjusting for age, sex, and income level. We estimated the observed to expected ratios (O/E ratios) for each variable value in each cluster by dividing the value of each variable in each cluster by the corresponding value in the entire study population. If the O/E ratio was ≥ 2, the variable was considered to clearly show the distinct characteristics of the cluster^41^. To evaluate the discrimination and stability of clusters in the study population, we calculated the exclusivity as the proportion of patients included in each cluster among all patients corresponding to each variable. After clustering, validation analysis was performed using the study sample that was reserved for the test set.

Statistical significance was set at *P* values of < 0.05, and all statistical analyses were performed using SAS version 9.4 (SAS Institute Inc., Cary, NC, USA).

## Supplementary Information

Supplementary Tables.

## Data Availability

Data are accessible from NHIS database, but the access to data used in this study is only available for the researchers who have applied for and have been granted. Further information is available in online homepage of National Health Insurance Sharing Service (https://nhiss.nhis.or.kr).
