# Supplementary material for: Patterns of patients with polypharmacy in adult population from Korea
Source: Sci Rep. 2022 Oct 27;12:18073. doi: 10.1038/s41598-022-23032-z (PMC9613698; doi:10.1038/s41598-022-23032-z)
Supplement: Supplementary file 1 — Supplementary Tables. [file 41598_2022_23032_MOESM1_ESM.pdf]

Patterns of patients with polypharmacy in adult population from Korea

Woo-young Shin, Tae-Hwa Go, Dae Ryong Kang, Sei Young Lee, Won Lee, Seonah Kim, Jiewon Lee, and Jung-ha Kim

Supplementary Table S1. Multiple linear regression analysis of health care utilization in six clusters of study patients with polypharmacy (cluster 6 as reference)[a]

Variable	Cluster 1			Cluster 2			Cluster 3			Cluster 4			Cluster 5		
	β	SE	P-value[b]	β	SE	P-value	β	SE	P-value	β	SE	P-value	β	SE	P-value
Number of prescribed medications per year	0.70	0.01	<0.001	1.10	0.01	<0.001	0.65	0.01	<0.001	0.76	0.01	<0.001	0.09	0.01	<0.001
Number of hospitalization days per year	4.11	0.01	<0.001	0.29	0.01	<0.001	− 0.37	0.01	<0.001	0.52	0.01	<0.001	0.08	0.01	<0.001
Number of outpatient visit days per year	5.28	0.11	<0.001	23.67	0.10	<0.001	24.23	0.12	<0.001	30.69	0.10	<0.001	0.96	0.11	<0.001
Number of visited medical institute per year	1.20	0.01	<0.001	2.06	0.01	<0.001	2.99	0.01	<0.001	4.38	0.01	<0.001	0.02	0.01	0.057

SE, standard error

[a] Adjusted for age, sex, and income level

[b] P-values <0.05 were statistically significant.

Supplementary Table S2. Observed/expected ratio and exclusivity for each variable in six clusters of the study population with polypharmacy

Variable	Observed to Expected ratio[a]						Exclusivity (%)[b]						P-value[c]
	Cluster 1	Cluster 2	Cluster 3	Cluster 4	Cluster 5	Cluster 6	Cluster 1	Cluster 2	Cluster 3	Cluster 4	Cluster 5	Cluster 6	
**Sex**													<0.001
Male	1.29	1.05	0.64	0.76	0.41	1.83	15.76	19.24	7.99	16.89	6.71	33.41	
Female	0.73	0.96	1.34	1.22	1.56	0.22	8.90	17.59	16.70	26.99	25.75	4.06	
**Age**													<0.001
30–39 years	1.30	0.85	0.75	0.35	0.00	2.81	15.94	15.67	9.36	7.68	0.00	51.36	
40–49 years	1.38	0.77	0.84	0.40	0.03	2.69	16.86	14.19	10.52	8.88	0.42	49.13	
50–59 years	1.31	0.80	1.09	0.59	0.37	1.99	15.96	14.79	13.65	13.13	6.04	36.44	
60–69 years	1.05	0.96	1.10	1.01	0.78	1.14	12.83	17.58	13.68	22.28	12.83	20.80	
70–79 years	0.79	1.18	0.94	1.42	1.25	0.26	9.70	21.71	11.71	31.42	20.72	4.75	
80–89 years	0.77	1.10	0.98	1.07	2.02	0.06	9.37	20.32	12.17	23.62	33.42	1.10	
≥90 years	0.64	0.86	0.81	0.58	3.24	0.00	7.85	15.73	10.16	12.76	53.51	0.00	
**Type of medical coverage**													<0.001
National Health Insurance subscriber	1.00	0.88	0.63	1.13	1.11	1.12	12.24	16.17	7.84	25.00	18.27	20.47	
Medical Aid beneficiary	0.99	1.92	3.82	0.00	0.20	0.09	12.13	35.23	47.68	0.00	3.25	1.72	
**Income level**													<0.001
Quarter 1	1.08	0.95	2.43	0.40	0.69	1.03	13.21	17.40	30.35	8.82	11.32	18.90	
Quarter 2	1.17	0.82	0.92	0.84	0.89	1.40	14.33	15.16	11.53	18.57	14.77	25.63	

Variable	Observed to Expected ratio[a]						Exclusivity (%)[b]						P-value[c]
	Cluster 1	Cluster 2	Cluster 3	Cluster 4	Cluster 5	Cluster 6	Cluster 1	Cluster 2	Cluster 3	Cluster 4	Cluster 5	Cluster 6	
Quarter 3	1.06	0.86	0.01	1.33	1.14	1.26	13.02	15.73	0.08	29.40	18.77	23.00	
Quarter 4	0.85	0.89	0.00	1.50	1.39	0.95	10.36	16.29	0.00	33.06	22.91	17.38	
**Disabled**	0.67	4.72	0.00	0.00	0.09	0.19	8.21	86.82	0.01	0.02	1.42	3.53	<0.001
**Number of visit days per year: outpatient**													<0.001
0–10 days	1.64	0.56	0.03	0.00	1.64	2.30	20.11	10.26	0.42	0.00	27.07	42.15	<0.001
11–20 days	1.33	0.91	0.46	0.15	1.67	1.66	16.24	16.77	5.79	3.32	27.59	30.29	
21–30 days	1.10	1.01	1.33	0.84	1.18	0.73	13.40	18.49	16.60	18.66	19.49	13.36	
≥31 days	0.42	1.24	1.63	2.19	0.08	0.10	5.08	22.88	20.30	48.48	1.35	1.91	
Hospitalization experience	3.36	1.14	0.79	0.99	0.16	0.20	41.13	21.01	9.87	21.77	2.63	3.58	<0.001
**Number of hospitalization days per year: inpatient**													<0.001
No hospitalization	0.00	0.94	1.09	1.01	1.36	1.34	0.00	17.28	13.59	22.23	22.39	24.51	<0.001
1–5 days	2.75	1.28	0.89	1.11	0.18	0.22	33.65	23.60	11.09	24.63	2.98	4.05	
≥6 days	8.02	0.07	0.05	0.00	0.00	0.00	98.03	1.37	0.60	0.00	0.00	0.00	
**Visited medical institute**													
Medical clinic	0.89	0.99	1.09	1.11	0.96	0.93	10.87	18.19	13.58	24.51	15.81	17.05	<0.001
Hospital	1.34	1.08	1.08	1.31	0.60	0.62	16.44	19.88	13.47	28.95	9.94	11.33	<0.001
General hospital	1.27	1.11	1.00	1.11	0.77	0.77	15.56	20.42	12.54	24.52	12.80	14.15	<0.001
Tertiary hospital	1.26	1.06	0.79	1.16	0.81	0.89	15.42	19.41	9.82	25.57	13.43	16.34	<0.001

Variable	Observed to Expected ratio[a]						Exclusivity (%)[b]						P-value[c]
	Cluster 1	Cluster 2	Cluster 3	Cluster 4	Cluster 5	Cluster 6	Cluster 1	Cluster 2	Cluster 3	Cluster 4	Cluster 5	Cluster 6	
Long-term care hospital	2.10	1.35	0.89	0.88	0.78	0.32	25.65	24.82	11.08	19.55	12.96	5.93	<0.001
**Number of visited medical institute per year**													<0.001
1 place	0.84	0.81	0.13	0.00	1.96	2.23	10.29	14.83	1.63	0.00	32.39	40.86	<0.001
2–5 places	1.29	1.02	0.93	0.33	1.40	1.30	15.73	18.71	11.56	7.20	23.07	23.73	
≥6 places	0.52	1.02	1.37	2.49	0.02	0.13	6.41	18.77	17.06	54.97	0.40	2.38	
**Diagnosed disease**													
Hypertension	0.93	1.01	0.97	1.03	1.07	0.96	11.41	18.49	12.11	22.66	17.69	17.64	<0.001
Dyslipidemia	0.97	0.92	1.00	1.06	0.94	1.08	11.88	16.94	12.49	23.42	15.60	19.67	<0.001
Knee arthrosis	0.69	1.09	1.37	1.58	0.84	0.31	8.42	20.11	17.04	35.01	13.82	5.60	<0.001
Diabetes mellitus	0.98	0.98	0.97	1.01	0.95	1.09	11.94	18.00	12.09	22.36	15.75	19.86	<0.001
Chronic ischemic heart disease	1.27	0.95	0.87	1.07	0.91	0.95	15.52	17.46	10.83	23.73	15.11	17.35	<0.001
Liver disease	1.17	0.92	1.16	1.11	0.59	1.10	14.26	16.92	14.42	24.50	9.75	20.15	<0.001
Depression	1.11	1.11	1.29	1.36	0.66	0.48	13.55	20.41	16.13	30.15	10.98	8.77	<0.001
Asthma/ Chronic Obstructive Pulmonary disease	1.02	1.10	1.23	1.37	0.71	0.55	12.41	20.27	15.33	30.18	11.66	10.14	<0.001
Osteoporosis	0.71	1.08	1.37	1.53	1.07	0.16	8.71	19.94	17.10	33.70	17.66	2.88	<0.001
Kidney disease	1.30	2.27	0.58	0.64	0.57	0.63	15.94	41.70	7.21	14.09	9.46	11.60	<0.001
Chronic stroke	1.29	1.44	0.82	0.93	0.97	0.60	15.82	26.40	10.22	20.55	16.08	10.93	<0.001

Variable	Observed to Expected ratio[a]						Exclusivity (%)[b]						P-value[c]
	Cluster 1	Cluster 2	Cluster 3	Cluster 4	Cluster 5	Cluster 6	Cluster 1	Cluster 2	Cluster 3	Cluster 4	Cluster 5	Cluster 6	
Dementia	1.28	1.36	1.01	1.01	1.26	0.19	15.66	24.97	12.60	22.33	20.89	3.55	<0.001
Anxiety disorder	0.91	1.06	1.29	1.49	0.67	0.49	11.15	19.55	16.14	32.99	11.12	9.05	<0.001
Parkinson's disease	1.37	1.66	0.81	0.94	0.88	0.40	16.77	30.48	10.13	20.73	14.59	7.30	<0.001
Cancer	1.74	1.00	0.97	1.25	0.64	0.56	21.24	18.44	12.09	27.55	10.50	10.18	<0.001
Chronic gastritis/gastroesophageal reflux disease	0.99	1.01	1.10	1.15	0.89	0.84	12.14	18.61	13.77	25.39	14.66	15.44	<0.001

[a] Observed to expected ratios were estimated for each variable value in each cluster by dividing the value of each variable in each cluster by the corresponding value in the entire study population. If the O/E ratio was ≥2, the variable was considered to clearly show distinct characteristics according to the cluster.

[b] Exclusivity was calculated as the proportion of individuals included in each cluster among all study patients corresponding to each variable to evaluate the distinction and stability of clusters in the study population.

[c] P-values of <0.05 were statistically significant.
